# Rare Cause of Wide QRS Tachycardia

**DOI:** 10.1155/2015/151269

**Published:** 2015-12-15

**Authors:** Nikolay Yu. Mironov, Natalia A. Mironova, Marina A. Saidova, Olga V. Stukalova, Sergey P. Golitsyn

**Affiliations:** ^1^Department of Clinical Electrophysiology, Russian Cardiology Research Center, Russia; ^2^Department of Sonography, Russian Cardiology Research Center, Russia; ^3^Department of Tomography, Russian Cardiology Research Center, Russia

## Abstract

Cardiac involvement is a well-known feature of neuromuscular diseases. Most commonly cardiac manifestations occur later in the course of the disease. Occasionally severe cardiac disease, including conduction disturbances, life-threatening arrhythmias, and cardiomyopathy, with its impact on prognosis, may be dissociated from peripheral myopathy. We report a case of bundle branch reentrant ventricular tachycardia as primary manifestation of myotonic dystrophy and discuss associated diagnostic and treatment challenges.

## 1. Introduction

Myotonic dystrophy (MD) is a rare genetic progressive neuromuscular disease. The prevalence of MD in general population is 1 : 8000 [1]. MD affects skeletal muscle resulting in increased muscular tonus (myotonia) and progressive muscular weakness. Multiple organs are also involved. Disease is associated with significant morbidity and mortality. Respiratory failure and cardiovascular pathology were the most prevalent causes of death, accounting for about 40% and 30% of fatalities, respectively. Cardiac mortality occurs because of progressive left ventricular dysfunction, ischaemic heart disease, or pulmonary embolism or as a result of sudden death [2]. Commonly cardiac involvement develops later in the course of disease in patients with previously established diagnosis and prominent neuromuscular symptoms. Occasionally, cardiac involvement may be the first sign of MD [3]. We report a case of bundle branch reentrant VT as primary manifestation of MD and discuss diagnostic and treatment challenges.

## 2. Case Presentation

A 45-year-old male with no previous cardiac disease and unremarkable familial history was admitted due to recurrent hour-lasting episodes of chest pain that was caused by wide QRS tachycardia that required DC cardioversion.

Upon admission patient was oriented, in no acute distress, well developed, and well nourished (body mass index 30 kg/m^2^). He denied smoking, alcohol abuse, and illicit drug use. Physical examination revealed hyperhidrosis. Patient's axillar temperature was 36.4°C (97.5°F), blood pressure was 110/80 mmHg on both arms, pulse was 86 bpm with a regular rhythm, and respiratory rate was 18 breaths/min. Chest and abdomen investigation was unremarkable. Extremities were warm and well perfused, with normal range of motion and no edema. Slight binocular ptosis and moderate peripheral muscle weakness were noted. Patient had no severe cognitive defects.

Clinical blood, thyroid, and coagulogic profiles were normal. Biochemistry panel revealed hyperlipidemia (total cholesterol 6.5 mmol/L [253 mg/dL], LDL-cholesterol 3.8 mmol/L [148.3 mg/dL]), and modestly elevated creatine kinase 347 U/L. Troponin test was negative. HbA1c level was 6.1%. BNP level was less than 10 pg/mL.

**Figure 1:**
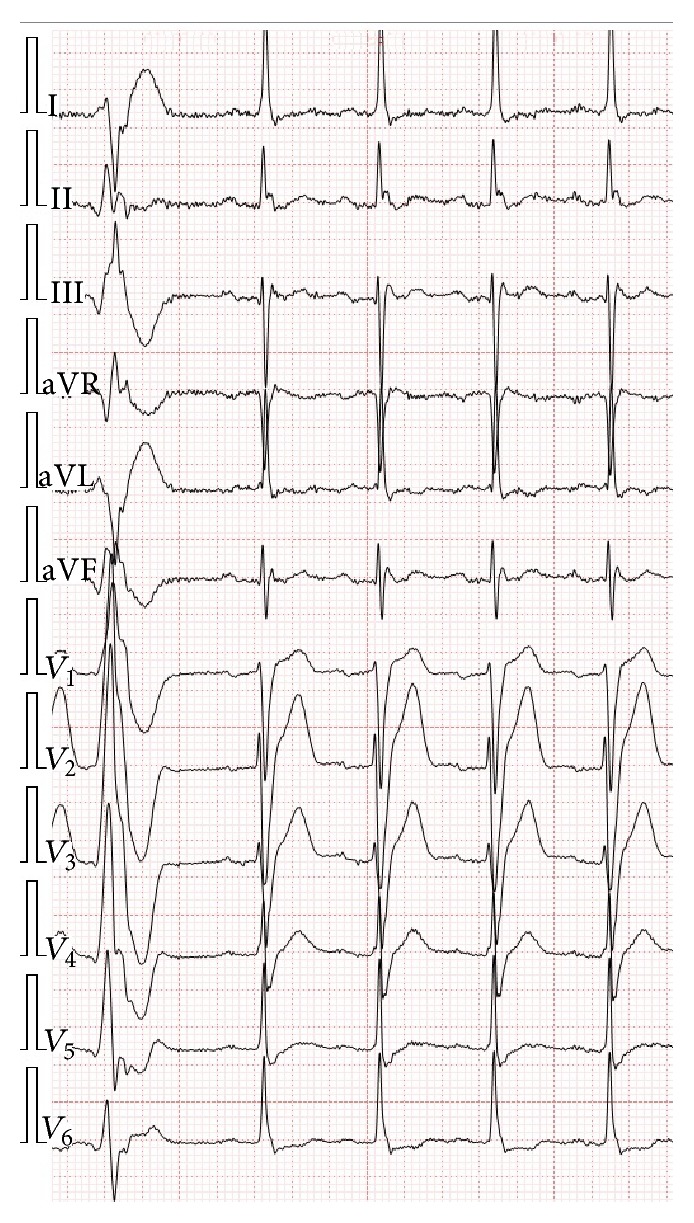
ECG at rest. Sinus rhythm 94 bpm, single premature ventricular complexes. Signs of left ventricular hypertrophy and atrioventricular (PQ 220 ms) and interventricular conduction abnormalities (QRS 120 ms). Recorded at 25 mm/sec, 10 mm/mV.

ECG at rest displayed sinus rhythm (95 bpm), PQ (200 ms), QRS (120 ms), and single premature ventricular complexes (Figure 1). 24-hour ECG monitoring registered 524 single premature ventricular beats with no significant sustained arrhythmias and pauses. Echocardiography revealed asymmetric nonobstructive hypertrophy of ventricular septum (up to 16 mm in basal segment) with preserved LVEF, no wall motion abnormalities, and enlarged LA (4.4 cm; volume 90 mL).

**Figure 2:**
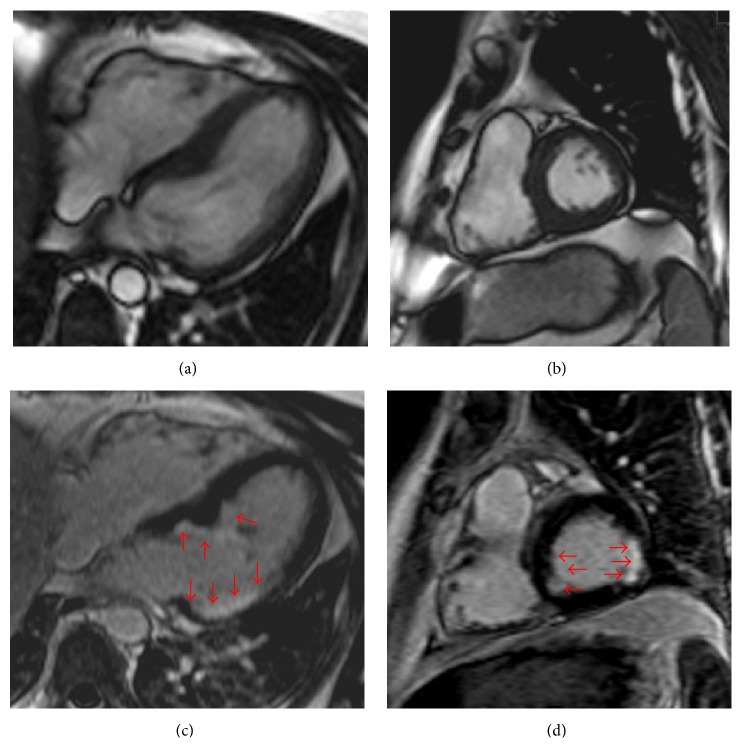
Cardiac magnetic resonance. Cine images in four-chamber long-axis view (a) and short-axis view (b). Late gadolinium enhancement (LGE) images in four-chamber long-axis view (c) and in short-axis view (d). Arrows point to regions of increased signal intensity, indicating focal fibrosis, visible as subendocardial enhancement.

Cardiac MRI demonstrated areas of subendocardial late gadolinium enhancement in septal, inferior, and lateral walls of LV, indicating focal fibrosis (Figure 2). Although subendocardial accumulation is typical for ischemic lesions, coronary angiography revealed intact arteries.

**Figure 3:**
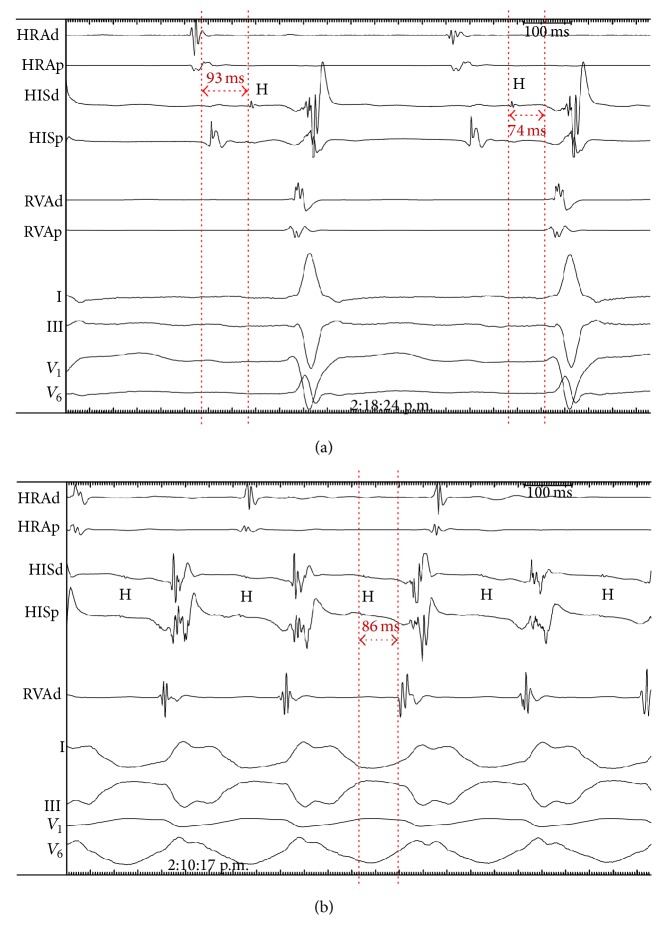
Results of electrophysiological investigation: (a) sinus rhythm; His bundle electrogram shows normal AH interval (93 ms) and delayed conduction in His-Purkinje system (HV interval 74 ms); (b) Bundle branch reentrant ventricular tachycardia: VA dissociation points to localization of reentrant circle within ventricles; typical bundle branch reentrant tachycardia features are (1) His bundle potential (H) precedes QRS in every beat and (2) HV interval in LBBB-shaped tachycardia (86 ms) is longer than HV interval on sinus rhythm. Tachycardia uses RBB as anterograde limb and LBB as retrograde limb of reentrant circuit; HRA, high right atrium; RVA, right ventricle apex; HIS, His bundle electrogram.

Electrophysiologic study showed delayed conduction in His-Purkinje system (HPS). HV interval duration was 74 ms (Figure 3(a)). Right ventricle pacing repeatedly induced bundle branch reentrant VT with a rate of 250 bpm and RBBB morphology with anterograde conduction over RBB and retrograde conduction over LBB (Figure 3(b)). All episodes of VT very successfully terminated by burst pacing. Considering structural heart disease and HPS involvement the decision was to refrain from radiofrequency ablation (RFA) and to implant 2-chamber ICD.

**Figure 4:**
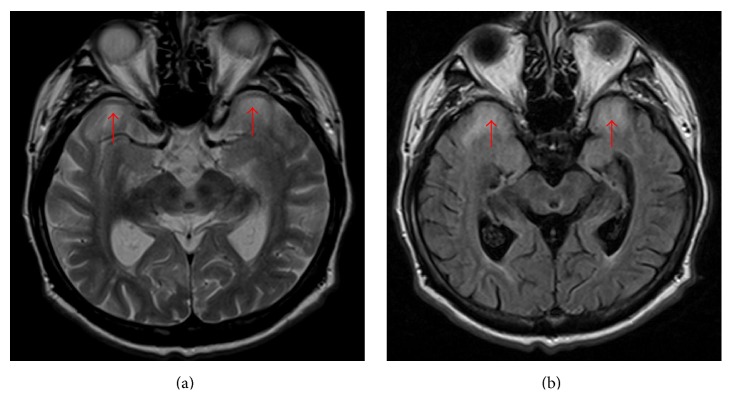
Brain MRI. Anterior temporal lobe hyperintensities on T2-weighted (a) and FLAIR (b) images.

Brain MRI prior to implantation showed multiple vascular lesions in white matter and anterior temporal lobe hyperintensities on T2-weighted and FLAIR images (Figure 4).

Patient was discharged on Bisoprolol 7.5 mg OD and referred to neurological center for further investigation where diagnosis of myotonic dystrophy (MD) was confirmed by electromyography. Genetic test revealed multiple CTG repeats in DMPK gene.

A 10-month follow-up was remarkable for significant progression of neuromuscular symptoms. However there were no signs of heart disease progression on ECG and echocardiography. ICD telemetry showed 4 appropriate shocks delivered to one episode of fast VT and multiple long-lasting episodes of Afib with mean ventricular rate of 86 bpm that were asymptomatic and diagnosed only at ICD interrogation. Considering hypertrophic cardiomyopathy anticoagulant therapy by rivaroxaban 20 mg OD was initiated.

We desired to refrain from RFA procedure again. ICD was reprogrammed to more aggressive antitachycardial pacing by addition of 4 extra burst pacing packs of shorter cycle length. In subsequent 8-month follow-up there were 3 sustained fast VT detections. All of them were terminated by burst pacing and were asymptomatic.

## 3. Discussion

Myotonic dystrophy type (MD) is the most common muscular dystrophy in adults. There are 2 forms of disease: MD type 1 (caused by expansion of a CTG trinucleotide repeat in the 3′-untranslated region of the dystrophia myotonica protein kinase gene [DMPK gene]) and MD type 2 (caused by an expanded CCTG tetranucleotide repeat expansion located in intron 1 of the zinc finger protein 9 gene [ZNF9]). Exact proportions in prevalence of MD type 1 and MD type 2 are unknown [1].

Cardiac involvement is frequent in both forms of MD but commonly affects patients with prominent neuromuscular symptoms [3, 4]. Cardiac disease is characterized by progressive conduction system abnormalities, supraventricular and ventricular arrhythmias, sudden death, and, less frequently, myocardial dysfunction (hypertrophic and, rarely, dilative cardiomyopathy) and ischaemic heart disease [5]. Conduction abnormalities that are of progressive course and potentially malignant may be found at any level of cardiac conduction system but commonly are located in HPS [4, 6]. In a study of 408 patients Groh et al. found that severe ECG abnormalities (rhythm other than sinus, PR interval of 240 ms or more, QRS duration of 120 ms or more, or second-degree or third-degree atrioventricular block) predict sudden death in type 1 MD [4]. ESC guidelines recommend pacemaker implantation if patient with MD develops any symptoms that may be caused by conduction system defect even if he has minor conduction abnormalities and does not meet classic pacemaker indications [7]. Tachyarrhythmias are also prevalent in MD patients. Most common arrhythmia is AFib. It is observed in up to 25% of patients in both sustained and nonsustained forms [4, 6, 8]. Since there are no data on risk of thromboembolic complications in that subset of patients, it is reasonable to use CHA_2_DS_2_-VASc score to define indications to anticoagulants. Malignant ventricular arrhythmias including monomorphic and polymorphic VT and spontaneous VF are also described. Delayed impulse conduction along HPS represents ideal substrate for bundle branch reentrant VT [9]. ICD is indicated in all patients with MD with sustained ventricular arrhythmias due to high risk of sudden death [4, 10]. RFA of RBB or LBB may be successfully applied in patients with bundle branch reentrant VT [1, 6].

Brain involvement is common, resulting in cognitive dysfunction, behavioral changes, apathy, and excessive daytime somnolence [1]. It should be noted that almost exclusively white matter lesions are found in MRI scans of patients with MD [11]. The origin of glious lesions in grey matter of both temporal lobes in our patient remained unknown.

In our case upon admission patient had only moderate neuromuscular symptoms but severe life-threatening ventricular arrhythmias that required ICD implantation. Furthermore, his familial history was unremarkable. Verification of uncommon cardiac lesions led to extensive diagnostic approach with suspicion of neuromuscular disease and subsequent referral to neurologic center, where the exact diagnosis of MD was made.

Taking into consideration small number of episodes of bundle branch reentrant VT, cardiac conduction system defects, and increased risk of LV dysfunction progression on permanent pacing, we desired to refrain from RFA, which is frequently referred to as method of choice in management of bundle branch reentrant VT. Thorough optimization of ICD antitachycardiac pacing parameters helped to terminate recurrent paroxysms. We did not initiate amiodarone therapy that time based on data that antiarrhythmic drugs rarely prevent bundle branch reentrant VT [9] and because of the risks of neurotoxicity that may be higher in patients with preexistent neurological disorders. We continued a close follow-up and if patient will have multiple episodes of bundle branch reentrant VT, either antiarrhythmic drug therapy might be initiated or he might be readmitted for RFA procedure. It should be noted that there was no significant cardiac disease progression in 18-month follow-up which could be frequently observed in patients with LBBB after bundle branch reentrant VT ablation [12].

Despite hyperlipidemia and high cardiometabolic risk, decision to refrain from statin prescription was made, powered by transient creatine kinase elevations and observations that patients with muscular metabolic abnormalities are more prone to statin-induced myopathy [13].

In the case described patient developed AFib significantly later than VT in the course of the disease. From the onset of the first paroxysm there is clear trend in AFib progression to permanent form. Although AFib was generally asymptomatic, it was clinically significant and required anticoagulation taking into consideration the presence of significant LV septal hypertrophy and high risk of cardioembolic events in patients with hypertrophic cardiomyopathy and AFib [14, 15]. Noteworthy, in this case CHADS_2_ and CHA_2_DS_2_-VASc scales were useless as the patient's score was zero.

## 4. Conclusion

Our case illustrates that cardiac arrhythmias in patients with MD may be more severe than neuromuscular symptoms, and their management could be challenging. Stepwise approach should be applied. Thorough consideration of pros and contras of any therapeutic modality is mandatory because these patients may not benefit from routine treatment strategies.
